# Primary care physicians’ perceived barriers and facilitators to conservative care for older adults with chronic kidney disease: design of a mixed methods study

**DOI:** 10.1186/s40697-016-0110-0

**Published:** 2016-04-04

**Authors:** Helen Tam-Tham, Brenda Hemmelgarn, David Campbell, Chandra Thomas, Robert Quinn, Karen Fruetel, Kathryn King-Shier

**Affiliations:** Department of Community Health Sciences, Cumming School of Medicine, University of Calgary, Calgary, Alberta Canada; Department of Medicine, Cumming School of Medicine, University of Calgary, Calgary, Alberta Canada; Faculty of Nursing, University of Calgary, Professional Faculties, Room 2209, 2500 University Drive NW, Calgary, Alberta T2N 1N4 Canada

**Keywords:** Chronic kidney disease, Conservative care, Mixed methods, Non-dialysis care, Older adults, Primary care physicians

## Abstract

**Background:**

Guideline committees have identified the need for research to inform the provision of conservative care for older adults with stage 5 chronic kidney disease (CKD) who have a high burden of comorbidity or functional impairment. We will use both qualitative and quantitative methodologies to provide a comprehensive understanding of barriers and facilitators to care for these patients in primary care.

**Objectives:**

Our objectives are to (1) interview primary care physicians to determine their perspectives of conservative care for older adults with stage 5 CKD and (2) survey primary care physicians to determine the prevalence of key barriers and facilitators to provision of conservative care for older adults with stage 5 CKD.

**Design:**

A sequential exploratory mixed methods design was adopted for this study. The first phase of the study will involve fundamental qualitative description and the second phase will be a cross-sectional population-based survey.

**Setting:**

The research is conducted in Alberta, Canada.

**Participants:**

The participants are primary care physicians with experience in providing care for older adults with stage 5 CKD not planning on initiating dialysis.

**Methods:**

The first objective will be achieved by undertaking interviews with primary care physicians from southern Alberta. Participants will be selected purposively to include physicians with a range of characteristics (e.g., age, gender, and location of clinical practice). Interviews will be recorded, transcribed verbatim, and analyzed using conventional content analysis to generate themes. The second objective will be achieved by undertaking a population-based survey of primary care physicians in Alberta. The questionnaire will be developed based on the findings from the qualitative interviews and pilot tested for face and content validity. Physicians will be provided multiple options to complete the questionnaire including mail, fax, and online methods. Descriptive statistics and associations between demographic factors and barriers and facilitators to care will be analyzed using regression models.

**Limitations:**

A potential limitation of this mixed methods study is its cross-sectional nature.

**Conclusions:**

This work will inform development of clinical resources and tools for care of older adults with stage 5 CKD, to address barriers and enable facilitators to community-based conservative care.

**Electronic supplementary material:**

The online version of this article (doi:10.1186/s40697-016-0110-0) contains supplementary material, which is available to authorized users.

## ABRÉGÉ

**Mise en contexte:**

Les comités pour l’établissement de lignes directrices ont relevé la nécessité pour la recherche de faciliter la mise en place d’un traitement conservateur chez les adultes âgés atteints d’insuffisance rénale chronique (IRC) de stade 5, des patients présentant une incidence élevée de comorbidité et d’altération de la fonction rénale. Des méthodes qualitatives et quantitatives seront utilisées pour assurer une compréhension détaillée des obstacles aux soins de première ligne et en contrepartie, des éléments qui les facilitent.

**Objectifs de l’étude:**

Cette étude vise deux objectifs principaux :

1) Interroger les médecins qui prodiguent des soins primaires afin de connaître leur point de vue sur l’administration d’un traitement conservateur chez les patients âgés atteints d’IRC de stade 5.

2) Sonder ces mêmes médecins pour établir la prévalence tant des obstacles à fournir des traitements conservateurs que des éléments la facilitant chez les patients âgés atteints d’IRC de stade 5.

**Cadre et type d’étude:**

Il s’agit d’un modèle d’étude exploratoire à méthodes mixtes qui sera effectuée de façon séquentielle. Une première phase impliquera une description qualitative fondamentale tandis qu’une deuxième consistera en une enquête transversale menée dans la population. Les deux phases de l’étude se tiendront en Alberta, au Canada.

**Participants:**

Un groupe de médecins prodiguant des soins primaires à des patients âgés atteints d’IRC de stade 5, mais n’envisageant pas de commencer des traitements d’hémodialyse.

**Méthodologie:**

Le premier objectif sera atteint en menant une enquête auprès des médecins du sud de l’Alberta prodiguant des soins de première ligne. Les participants seront sélectionnés avec l’intention d’inclure des praticiens représentant un éventail de caractéristiques (âge, genre, lieu de pratique). Les entrevues seront enregistrées puis transcrites intégralement, et l’information recueillie sera traitée en utilisant une approche conventionnelle d’analyse du contenu pour en tirer les thématiques. Le second objectif sera également réalisé en procédant à une enquête dans la population de médecins prodiguant des soins de première ligne en Alberta. Le questionnaire sera mis au point suivant les résultats obtenus lors des entretiens qualificatifs et testés lors d’essais-pilotes visant à établir leur validité apparente et de contenu. Les praticiens disposeront de plusieurs moyens pour retourner le questionnaire une fois complété soit par la poste, par fax ou par d’autres méthodes en ligne. Les données statistiques descriptives ainsi que les associations établies entre les facteurs démographiques et les obstacles et facilitateurs de soins seront analysées à l’aide de modèles de régression.

**Limites de l’étude:**

Nous prévoyons une éventuelle limite à cette étude par méthodes mixtes compte tenu de sa nature transversale.

**Conclusions:**

À terme, ces travaux orienteront le développement de ressources cliniques et d’outils pour la prise en charge des patients âgés atteints d’IRC de stade 5. Ils contribueront également à rendre possible l’établissement de programmes de traitements conservateurs communautaires et à éliminer les obstacles rencontrés.

## What was known before

Many patients with stage 5 chronic kidney disease that are not treated with renal replacement therapy are often provided by primary care physicians, many independently without consultation from nephrology. Providing conservative care in the primary care setting is complex and incompletely understood.

## What this adds

This paper presents a mixed methods study to comprehensively understand barriers and facilitators to community-based kidney conservative management by primary care physicians, which can serve as an example for other investigators interested in mixed methods research within the field of nephrology.

## Background

There is a large proportion of older adults with stage 5 chronic kidney disease (CKD) not treated with renal replacement therapy. In Alberta, Canada, 51 % of people with stage 5 CKD not treated with renal replacement therapy are aged 75 years and older [1]. Individuals with stage 5 CKD often live with multiple morbidities and poor life expectancy. Conservative management is a non-dialysis treatment option that is chosen by patients or is medically advised [2]. It encompasses a planned holistic patient-centered approach including interventions to delay CKD progression, symptom management, advance care planning, psychological support, and family support [2]. Clinical practice guidelines suggest that this management strategy should include a comprehensive program with coordinated end-of-life care for patients and their families via primary or specialist care [3]. Nevertheless, there is a need to optimize the provision of conservative care in this particularly vulnerable older adult population with stage 5 CKD, as these programs are underdeveloped in many locations [4]. Previous research on the provision of conservative management programs have identified variations in renal unit specific guidelines and the availability of dedicated conservative care staff and training [5].

Approximately 40 % of patients with stage 5 CKD not treated with renal replacement therapy have not been seen by a nephrologist within a 2-year period [1]. Hence, conservative care is often provided by primary care physicians, many independently without consultation from nephrology, who are also the first points of contact with the healthcare system for the majority of day-to-day healthcare needs. However, providing conservative care in the primary care setting is complex and incompletely understood. By understanding barriers and facilitators to community-based conservative management by primary care physicians, potential interventions can be developed to support the development, accessibility, and quality of conservative management programs where they are needed. The research objectives in this program of study aim to yield information that can be integrated into routine decision-making processes for professionals who care for older adults with stage 5 CKD. The first objective is to describe primary care physicians’ perceived key barriers, facilitators, and strategies to enhancing conservative care for older adults with stage 5 CKD. The second objective is to determine the prevalence of key barriers and facilitators to conservative care gathered from the first objective.

## Methods/design

### Mixed methods study design

**Fig. 1:**
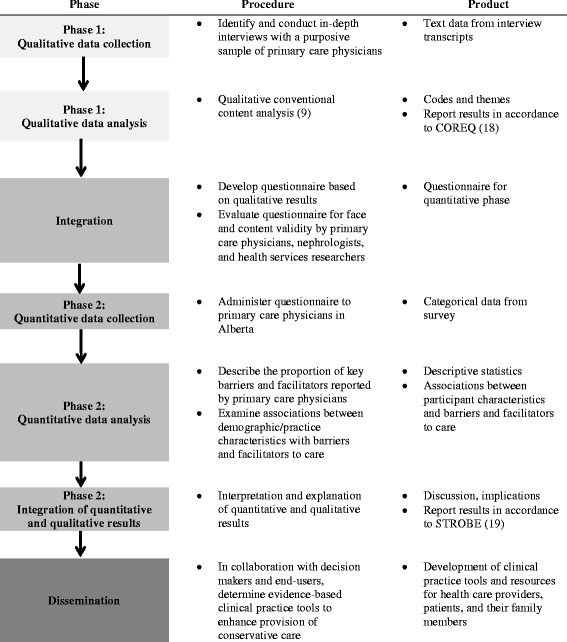
The mixed methods study design

Mixed methods research involves the following: identification of a specific mixed method research design; data collection and analysis of qualitative and quantitative data; integration of the two forms of data; and assignment of prioritization to either or both forms of data [6]. A sequential exploratory mixed methods design (Fig. 1), a two-phase study design, will be conducted for the purposes of this study. The first phase of the study will involve qualitative description using interviews to understand and describe primary care physicians’ perspectives of care for older adults (aged 75 years and greater) with stage 5 CKD (estimated glomerular filtration rate (eGFR) less than 15 mL/min/1.73 m^2^ for at least 3 months) not on dialysis in the primary care setting. A subsequent quantitative phase (phase 2) will be designed to quantify and describe the magnitude of barriers and facilitators to conservative care. The survey will be used to also examine the relationship between demographic and clinical practice characteristics and barriers and facilitators to care. The quantitative phase will involve questionnaire design, testing, and administration. The qualitative and quantitative phases are assigned equal priority and the two phases will be integrated sequentially (i.e., the quantitative phase will build on the qualitative phase). A study in the field of nephrology using a similar type of design has been previously reported [7]. Ethical approval of this mixed methods study has been obtained from the Conjoint Health Research Ethics Board at the University of Calgary.

### Phase 1: qualitative interviews

#### Qualitative paradigm

Primary care physicians’ perspectives of barriers and facilitators to caring for older adults with stage 5 CKD are complex and incorporate individual views and experiences of diagnosis, treatment, and referral. Hence, the nature of this research question encompasses multiple truths and rich perspectives. These multiple perspectives are best captured through the collection of data in the natural setting or context where the participants experience the barriers and facilitators [8]. Ultimately, the qualitative paradigm is ideal for this component of the research, as it allows for a comprehensive and nuanced account of the problem under study. Fundamental qualitative description [9] will be employed to develop a comprehensive summary of barriers and facilitators experienced by physicians when providing care to older adults with stage 5 CKD.

#### Participant selection and recruitment

Using a snowball sampling approach, key stakeholders or opinion leaders in areas of family medicine, seniors’ health, and kidney disease will be asked to identify primary care physicians with experience caring for older adults with stage 5 CKD in the community. Eligible participants will include physicians who currently practice in the primary care setting, have at least 1 year of experience as a primary care physician, and have experience caring for patients 75 years or older with stage 5 CKD in the past year. Consenting physicians will be interviewed in-person or via telephone, based on their preference. Participants will also be asked to recommend other providers who meet the eligibility criteria.

#### Data collection

Participants will be asked to provide basic information regarding demographics and practice characteristics. An interview guide has already been developed based on a review of the literature and discussion with the research team, including key decision-makers. The broad introduction question, probing questions, and closing question will be pilot tested with three primary care physicians (see Additional file 1). The interview guide considered barriers, facilitators, and strategies regarding the following overarching topics: stage 5 CKD diagnosis and assessment, care management, and resource use. One interviewer (HT) will conduct the interviews to ensure consistency in interview style and structure. Participant recruitment will cease when theoretical saturation is reached (i.e., when new information or concepts no longer emerge from additional interviews). All interviews will be audio-recorded and then transcribed verbatim by a professional transcriptionist.

During the semi-structured interviews, providers will be asked about their perspectives on caring for the population of interest. The probing questions are designed to help interviewees think about barriers they experience regarding diagnosis, care management, and referral decisions, should these topics not develop spontaneously. Participants may also be asked about potential strategies or facilitators they could employ to address their challenges. All participants will be asked the broad introductory and closing questions, with probing questions used only when necessary. Field notes will also be documented on the participants’ responses to interview questions, which may inform data analysis and subsequent conduct of interviews.

#### Qualitative analysis

Perceptions relating to diagnosis and management decision-making will be identified and categorized using conventional content analysis [10], a method of interpreting interview data with the goal of describing the phenomenon of interest. The steps for conventional content analysis include the following: (1) achieving immersion by first reading the interview data in its entirety to acquire an overall sense of the phenomenon; (2) reading interviews word by word and highlighting words that capture key concepts that become codes; (3) documenting initial impressions, thoughts, and interpretation; (4) developing codes; and (5) sorting codes that are related to each other into themes and sub-themes. Definitions will be developed for existing codes, themes, and sub-themes, and exemplars of these will be reported in the findings. Coding will be conducted in triplicate (i.e., there will be three independent researchers coding each of the manuscripts) and then reviewed with the research team.

The process will be reflexive and interactive as continual data collection and data analysis will shape each other [9]. For example, code titles or definitions identified based on the first interview may be modified based on data collected during the second interview, and new codes may be added requiring recoding of the first interview transcript. Codes will be generated from the interview data, and they will be systematically applied to identify themes and patterns. Results from the qualitative phase of this mixed methods study has been published elsewhere [11]. These results will facilitate item generation for survey development in the next study phase, as the codes, themes, and sub-themes will directly form the variables of interest in the questionnaire (phase 2).

### Phase 2: quantitative survey

#### Participant selection

The sampling frame for this population-based survey will include all primary care physicians from the College of Physicians and Surgeons of Alberta, a provincial regulatory body of medical practice for physicians [12]. Employing a series of screening questions on the cover letter of the questionnaire, physicians will be asked to complete the questionnaire only if they recall ever having experience providing care to people 75 years or greater with an eGFR of less than 15 mL/min/1.73 m^2^. Providers will be invited to participate via mail, fax, and online methods.

#### Questionnaire development and administration

Based on results from the qualitative phase of the study, the questionnaire will obtain more information about barriers and facilitators to provision of care for older adults with stage 5 CKD in the community and will include a combination of multiple choice, Likert scales, and open-ended responses. The questionnaire is likely to reflect diagnosis, management, and referral decision-making components of care for older patients with stage 5 CKD. Potentially sensitive items focused on personal attributes and clinical practice settings will be situated at the end of the questionnaire and will include the following: the respondent’s age; gender; practice interests (e.g., care of the elderly) [13]; years practicing in this specialty; time allocated for clinical practice; and practice in an urban or rural area based on population. Previous studies have demonstrated that personal attributes and practice environments are key predictors of behavior among primary care physicians [14]. The questionnaire will be developed in two steps: item identification and preliminary development of the questionnaire and questionnaire refinement.

#### Item identification and preliminary development of the questionnaire

Potential items for inclusion in the questionnaire will be informed via a literature review and interviews from phase 1. Hence, addressing the qualitative objective will provide specific items relating to primary care for the patient population of interest, which will be categorized into different domains according to the Theoretical Domains Framework [15, 16]. The Theoretical Domains Framework provides a strategy for assessing problems related to implementation of clinical practice guidelines and provides a foundation for intervention development. It has been validated and demonstrated to be useful over a range of different healthcare systems, containing the following 14 domains that may influence behavior and provide a comprehensive structure for categorizing barriers and facilitators to care: knowledge; skills; social/professional role and identity; beliefs and capabilities; optimism; beliefs about consequences; reinforcement; intentions; goals; memory, attention and decision processes; environmental context and resources; social influences; emotion; and behavioral regulation. All domains of the framework will be reviewed for inclusion during this stage based on their relevance to the research objective.

#### Refining the questionnaire

Face validity (i.e., extent to which the questionnaire appears to be measuring the intended information) and content validity (i.e., extent to which questionnaire has enough coverage of the content area or sufficient number of questions for each domain) [17] will be assessed. At least three primary care physicians, nephrologists, and health services researchers (*n* = 9) will independently review the questionnaire for face and content validity, to evaluate the merit (e.g., relevance and quality) of each item, to identify unnecessary or ambiguous questions (to assess clarity, relevance, flow, and wording), and to determine whether respondents will interpret the questions appropriately and consistently (to assess interpretation) [18]. The length of time required to complete the questionnaire will also be measured.

#### Survey administration

Approximately 4500 primary care physicians will be contacted through information publicly available from the College of Physicians and Surgeons of Alberta [13]. Potential participants will be recruited via mail, fax, and online methods. The first contact will be a survey package that includes a personalized cover letter with screening questions to ensure only eligible physicians complete the questionnaire, a succinct questionnaire, a return fax number and email address, and an online survey as another option for completing the survey. Based on a modified Dillman approach [19], the first reminder with the same information (via fax) will be sent 1 week, the second reminder 3 weeks (mail), and the third reminder 4 weeks (fax) after the initial contact for non-responders.

#### Quantitative analysis

Questionnaire responses will be reported using descriptive statistics on the proportion of key barriers and facilitators perceived by primary care physicians in Alberta. Associations between demographic and practice characteristics and outcome variables (e.g., self-reported familiarity and use of comprehensive conservative programs) will be analyzed using regression models with backward elimination techniques. Based on a sample size calculation, a sample size of 371 eligible respondents will produce a 95 % confidence interval equal to the sample proportion plus or minus 0.05 when the estimated proportion is 0.50. At the interpretation stage, the extent to which the quantitative results extend the initial qualitative findings will be discussed.

#### Dissemination

Employing an integrated knowledge translation approach, decision-makers (leaders in primary care as well as the renal programs) have been involved in the development of study design, including interview and survey questions. Results from this mixed methods study will be published in peer-reviewed scientific journals, published in provincial and local newsletters targeted to primary care providers, and presented at national and international conferences to audiences in the areas of nephrology, family medicine, and geriatrics. In collaboration with key stakeholders of providers in the conservative care of patients with stage 5 CKD, the findings of this study will be used to inform the development of clinical practice tools and resources, including a provincial conservative kidney management clinical care pathway as a standardized informational tool to implement clinical practice guidelines and best practices in renal care, for healthcare providers, patients, and their family members.

## Discussion

The focus of this study is on investigating a growing, yet relatively under-studied, population of older adults with stage 5 CKD managed by primary care physicians in the community. To our knowledge, this is the first study of primary care physicians’ perspective in Canada and will address key areas that have not been previously investigated: barriers and facilitators primary care physicians experience when providing care for this older patient population and the prevalence of challenges and enablers to conservative care experienced by these providers. Data and results from this work will inform the provision of care by healthcare providers for older adults with stage 5 CKD in the community, increase the awareness of provincial renal conservative care clinics and other related initiatives such as an online conservative kidney management pathway, and support the development of a collaborative network of healthcare providers and researchers focused on improving the conservative care processes, outcomes, and future areas of investigation.

A potential limitation of this mixed methods study is its cross-sectional nature. We do not anticipate opinions about barriers and facilitators to care to evolve substantially over time, though further investigation is warranted following intervention strategies to improve conservative care. Also, we focus on older adults with stage 5 CKD to provide a clearly defined patient population and acknowledge that younger adults may undergo conservative care as well. This specific subgroup of patient may present a challenge in recruitment, as not all providers will have the required experience.

This study protocol presents a rigorous yet feasible mixed methods study design that offers a valuable opportunity to obtain in-depth, rich data to explore perspectives of conservative care in the primary care setting (e.g., via purposive sampling to ensure diverse participation and triplicate coding and to ensure data quality). It also provides an opportunity to examine the magnitude of potential challenges and solutions reported by providers caring for this patient population (e.g., via a census survey based on a validated data collection approach and descriptive and statistical analytical approaches). Results based on this survey will likely be generalizable to other Canadian and similar contexts internationally with universal health care. Ultimately, this province-wide study will incorporate an integrated knowledge translation approach to ensure its clinical relevance, identify future interventions, and support quality of care by providers for older adults with chronic kidney failure residing in the community.

## Additional file

Additional file 1: The interview guide used to support individual interviews in the qualitative phase of the mixed methods study. (DOCX 128 kb)
