# Supplementary material for: Primary care physicians’ perceived barriers and facilitators to conservative care for older adults with chronic kidney disease: design of a mixed methods study
Source: Can J Kidney Health Dis. 2016 Apr 4;3:17. doi: 10.1186/s40697-016-0110-0 (PMC4819283; doi:10.1186/s40697-016-0110-0)
Supplement: Additional file 1: — The interview guide used to support individual interviews in the qualitative phase of the mixed methods study. (DOCX 128 kb) [file 40697_2016_110_MOESM1_ESM.docx]

**Supplementary Information: Interview Guide**

*Broad introduction question:*

- I would like to learn about your experience caring for older adults (age 75 years and greater) with severe CKD (eGFR <15mL/min/1.73m^2^). What was your experience like?

*Probing questions on diagnosis and assessment:*

- Are there any specific issues or challenges that you encountered with diagnosis or assessment of older adults with severe CKD?
  - Why is that challenging? Are there any specific issues? Can you give me an example? How do you deal with these challenges?

*Probing questions on care management:*

- Are there any challenges in caring for older adults with severe CKD in the community? These may be related to the patient, their caregivers, and/or the family involved in their care.
  - How do you deal with these challenges?
- Are there any challenges with symptom control?
  - Are there any challenges in managing nausea? Fatigue? Itching?
  - Are there any resources, tools, or strategies that help with treating these symptoms?
- Are there any challenges in managing pain?
  - Are there any resources, tools, or strategies that help with pain control?
- Are there any resources, tools, or strategies in general that you find helpful when caring for older adults with severe CKD?
- Do you have any suggestions that may help address the challenges that you identified or help improve care in the future for older adults with severe CKD by primary care in the community?
- (If they mentioned use of specialty services such as nephrology or palliative care in management of the elderly with severe kidney disease): You mentioned that you use nephrology services, how do you use them to help you manage these patients? How do you use palliative care services to help you management these patients?

*Probing questions on resource use:*

- What resources and services do you access to care for older adults with severe CKD?
- Hypothetically, in an ideal world with unlimited resources, what kind of services would be available or what improvements could be made to help you care of older patients with severe CKD?
- Are you aware that nephrology has a conservative care program that assists in the care of patients with severe kidney failure who do not want to initiate dialysis?

*Closing question:*

- Is there anything else you would like to add relating to caring for older adults with severe CKD?
